# The relationship between shift work, circadian rhythms, and cognitive function in ICU nursing

**DOI:** 10.1186/s12912-025-02850-2

**Published:** 2025-03-26

**Authors:** Saeideh Moosavi, Mehran Ghalenoei, Mohammad Amerzadeh, Ali Safari Variani

**Affiliations:** 1 Student Research Committee, School of Public Health, Qazvin University of Medical Sciences, Qazvin, Iran; 2 Social Determinants of Health Research Center, Research Institute for Prevention of Non-Communicable Diseases, Qazvin University of Medical Sciences, Qazvin, Iran; 3 Non-communicable Diseases Research Center, Research Institute for Prevention of Non- Communicable Diseases, Qazvin University of Medical Sciences, Qazvin, Iran; 4 School of Public Health, Qazvin University of Medical Sciences, Qazvin, Iran

**Keywords:** Circadian rhythm, Nurses, Cognitive performance, Attention, Working memory

## Abstract

**Background:**

Nurses work 24-hour shifts due to the critical importance of patient care. The human body has a circadian rhythm that regulates many physiological activities. Shift work is associated with decreased melatonin levels and disruption of the circadian rhythm. Given the vital role of cognitive functions such as working memory and attention, this study investigated the impact of circadian rhythm disturbances on the cognitive performance of intensive care unit (ICU) nurses in Qazvin City, Iran.

**Methods:**

The study population consisted of 36 nurses. The assessment tools included a demographic questionnaire, a circadian rhythm questionnaire, and cognitive function tests (attention and working memory) using the Stroop Test and Wechsler Adult Intelligence Scale, administered at the beginning and end of each of the three shifts. The data was analyzed using descriptive statistics, including mean, standard deviation, and frequency percentages. The Kolmogorov-Smirnov test was used to determine the normality of the data. The data analysis involved analytical statistics using mixed-effects models performed using R software version 4. The significance level was at *p* < 0.05 for the present study.

**Results:**

The nurses exhibited a high degree of circadian rhythm inflexibility, with 83.3% classified as " inflexibile” and 91.7% as " vigorous “.|The nurses’ cognitive performance was highest during the morning shift, with better attention and working memory abilities. A one-unit increase in the stability and amplitude of the circadian rhythm was associated with reductions in memory span, congruent response time, and incongruent response time during the evening and night shifts.

**Conclusion:**

Given the observed decline in specific cognitive functions during evening and night shifts, the likelihood of increased errors during these shifts is heightened. The cumulative effect of circadian rhythm disruptions can manifest as diminished cognitive performance. The rhythm stability and amplitude could serve as predictive indicators for staffing shift work systems to prevent errors and enhance the system’s efficiency.

## Introduction

Shift work is an important social phenomenon in the structure of various occupations. Shift work, which is common in industries such as oil, power generation, and steel production, has increasingly become an essential aspect of modern life, particularly within service sectors such as healthcare, firefighting, and related fields. Shift work, prevalent in industries like oil, power generation, steel, and iron, has become an integral part of human life in specific service sectors such as healthcare, firefighting, and others [1]. Physicians and other healthcare personnel are constantly on call based on a shift-work system [2]. According to statistics, around 50% of the healthcare workforce in Iran are nurses who require shift work more than any other occupation [3, 4].

Many nurses experience high levels of stress due to the nature of their work, which involves dealing with life-and-death situations. The around-the-clock hospital presence and rotating shifts lead to increased stress and sleep disturbances, with studies showing that over 57% of shift workers suffer from sleep disorders [5]. Nurses often complain of insomnia and fatigue symptoms during night shifts, as the circadian rhythm promotes sleep during this period. This naturally elevates the probability of errors, with estimates suggesting a 30% higher incidence compared to day shift personnel [6].

“Circadian rhythm " refers to the daily, 24-hour rhythmic changes in behavioral activities and metabolism of living organisms. Virtually all physiological functions, tissues, and cells in the human body are regulated by the circadian clock. The human body also has an inherent circadian rhythm that regulates many functions [7, 8]. Shift work is associated with decreased melatonin levels and disruption of the circadian rhythm [9]. Rotating shift schedules can lead to increased sleep disturbances and fatigue, elevated risk of gastrointestinal disorders, short-term neurological issues, as well as increased susceptibility to obesity and diabetes [10]. Shift work is also linked to impaired immune function, with shift workers often exhibiting weaker immune systems [11]. Disruption of the natural circadian rhythm can also impact an individual’s psychological well-being and social/family relationships [12]. In the long term, shift work may increase the risk of mental health disorders, particularly depression and anxiety [6].

Several studies have demonstrated the adverse effects of shift work on mental processes, which consequently impair both cognitive and executive performance [13–16].For instance,, one study [13], found that shift work is associated with reduced cognitive performance in areas such as processing speed, working memory, psychomotor vigilance, cognitive control, and visual attention. Similarly, another study [14] supports these findings by indicating that while shift work exerts a long-term negative impact on cognitive performance, this effect may be reversible upon cessation of shift work. These findings underscore the significant influence of sleep deprivation and circadian rhythm disruption on cognitive functioning, particularly among shift workers in healthcare settings.

Despite the many adverse effects of shift work on human health, the migration of people to cities, industrial and factory development, and economic needs have led to an increase in shift-based occupations [10]. Patient safety remains a critical challenge for healthcare systems today, with nursing errors representing a significant proportion of medical errors.The incidence rate of errors in healthcare systems globally is high, with one out of every ten patients experiencing a medical error, and approximately 7% of these errors being fatal [17, 18]. In Iran, the significance of medical error is evidenced by the legal prosecution of 51% of nurses [19].

Recent studies suggest that while certain personality traits are associated with incidents, many of these characteristics are primarily independent of each other. In all these studies, human factors have been identified as one of the most important contributors to occupational accidents, although work environment conditions and the nature of job tasks also play a role. Cognitive factors, which have recently gained the attention of psychologists, are among these human factors [20]. Cognitive failures are cognitive-based errors resulting from problems with memory, attention, and action, occurring during a simple task that a typical person should perform without mistake [21].

Recent studies related to safety have examined the impact of cognitive processes on accidents. A 2019 study by Petita et al. showed a positive correlation between the incidence of cognitive failures and accidents [22]. Even temporary reductions in mental efficiency can have serious personal and material consequences, especially during critical stages of work processes that require rapid and accurate responses. Studies have shown that sleep is one of the factors that can affect alertness and cognitive performance in work situations. Complete or partial sleep deprivation is sometimes accompanied by decreased cognitive performance [23].

Cognitive functions such as working memory and attention play a vital role in executing many tasks. Therefore, even temporary impairment in cognitive and mental performance can lead to serious consequences for individuals, especially when there is a need for accurate and immediate responses [24]. Decreased cognitive performance in the workplace, particularly during night shifts, is a primary concern for healthcare systems [25].

Studies on the impact of shift work on cognitive performance are controversial. Tadevinac et al. found that shift work does not harm short-term tasks requiring focus and working memory. It reduces performance on long-term, monotonous tasks [26]. Errors are a byproduct of human information processing or cognitive performance [27]. Therefore, individual differences in cognitive abilities can lead to different types and rates of errors that people make in similar situations [28]. Various studies have shown that cognitive failures play a major role in occupational performance and safety [29].

Human errors have multiple causes, but in all cases, cognitive abilities and human limitations play an important role. Cognitive failures are cognitive-based errors in performing routine and simple tasks that a person should complete without mistakes. These errors may occur in one or all three stages of information processing: memory, attention, and action [30, 31]. Arthur et al. examined the relationship between cognitive failures and workplace incidents, finding a positive correlation and concluding that many accidents occur due to inattention, distraction, and mental errors [32]. Wallace and Chen also found a positive correlation between cognitive failures and accidents and a negative correlation with safety behaviors [33]. Studies evaluating the relationship between cognitive failure and safety outcomes, focusing on types of cognitive failures, have shown that failures like memory lapses and inattention can predict individual performance and behavior [29, 34].

Many studies have demonstrated the negative impact of shift work on mental processes and, consequently, on cognitive and executive performance [16, 26]. However, there are some discrepancies in the existing literature.

The intensive care unit (ICU) is one of the vital components of hospitals, accommodating patients in critical condition who are at risk of mortality. This unit can restore the health of severely ill patients by providing appropriate medical services, optimally utilizing modern medical equipment, leveraging the expertise of qualified and competent staff, and through the team-based decision-making process regarding patient care [35]. The ICU can be likened to a small hospital within a larger one, as it provides life-saving care for patients in critical condition [36]. This unit is the control point for hospital performance, and adherence to standards in the ICU is of utmost importance, as the delivered services must align with predetermined requirements to achieve quality management goals [37]. Therefore, developing and implementing standards in ICUs leads to improved patient survival and cost savings [36].

Given that the ICU is one of the specialized and critical hospital departments dedicated to caring for patients who require life-sustaining support and are at a very high risk of organ failure and death, this study aims to to examine the relationship between shift work, circadian rhythms, and cognitive function among ICU nurses.

## Methods

### Study design

This is a descriptive-analytical cross-sectional study conducted in 2023 among shift-working nurses in the ICU of a hospital Qazvin. The data was collected over a four-month period, from May to August 2024.”

### Participants and recruitment

The samples included shift-working nurses who met the inclusion criteria: willing to participate, at least one year of work experience, employed in rotating shifts, and not currently enrolled in education or training programs. We obtained informed consent and excluded participants if they met any of the exclusion criteria: using sleep medications, history of psychiatric disorders, or using cardiovascular, antidepressant, sedative, hypnotic, or anti-Parkinson’s medications. The study population consisted of 36 nurses.Shift Start and End Times: “The three shifts included:

Morning shift: 7:30 AM − 2:00 PM.

Evening shift: 1:30 PM − 8:00 PM.

Night shift: 7:30 PM − 8:00 AM”.

Shift Rotation: “All nurses rotated through all three shifts according to a rotating shift schedule.

### Data collection

#### Demographic questionnaire

The demographic questionnaire included questions about age, gender, marital status, clinical work experience, department, work shifts, history of illness, and medication use. The researcher first provided the necessary explanations about the study. The participants subsequently submitted their self-reported information in the tranquil and supportive environment of the nursing supervisor’s office [38, 39].

#### Determination of Circadian Rhythm Status

The Persian version of the Circadian Type Inventory (CTI), developed in 2011 specifically for Iranian day and shift workers, was employed in the present study. The CTI evaluates two domains: circadian rhythm stability and circadian rhythm amplitude. The reported Cronbach’s alpha values indicating validity and reliability for the shift-working nurse population were 0.70 and 0.82, respectively [40]. The overall reliability of the questionnaire, as measured by Cronbach’s alpha, was 0.76.The CTI questionnaire has 11 questions covering two independent factors. The first factor, “Flexible/Rigid”, represents circadian rhythm stability. Individuals scoring high on this factor are considered flexible and capable of shift work, with the ability to stay awake at unusual times of the day or night. The second factor, “Languid/Vigorous”, represents circadian rhythm amplitude. Those with high scores on this factor are “languid”, finding it more difficult to overcome feelings of sleepiness and sluggishness due to sleep deprivation.

The questionnaire items are rated on a 5-point Likert scale (1 - almost never, 2 - rarely, 3 - sometimes, 4 - usually, 5 - almost always), with total scores ranging from 11 to 55.

Individuals who score above 18.75 out of 25 on the “Flexible/Rigid” factor of the CTI questionnaire are in the 75th percentile and have a flexible circadian rhythm. These individuals can stay awake at unusual times of the day or night.

Participants who score above 22.5 out of 30 on the “Languid/Vigorous” factor are also in the upper 75th percentile and have a languid circadian rhythm amplitude. For these individuals, overcoming feelings of sleepiness and sluggishness is more challenging.

To complete this questionnaire, the researcher first provided the necessary explanations to the participants. The participants then self-reported the information in the calm and conducive environment of the nursing supervisor’s office.

#### Evaluation of cognitive performance (selective attention)

Rapid and easy measurement of selective attention can provide valuable insights into an individual’s ability to focus. The Stroop test assesses frontal lobe function and evaluates selective attention. Macleod designed this test in 1996.

Stroop Test: “The Stroop test was administered using a computerized version developed in-house using.”

The test consists of three stages:

Preliminary: In this stage, the participant presses the button corresponding to the circle color displayed on the screen. The circles are in four colors: red, blue, yellow, and green. The purpose of this stage is only to practice and familiarize the participant with the colors and keys’ location on the keyboard, and it does not affect the final results.

Explanation: To reduce the test duration, the preliminary stages that did not affect the results were omitted from this study, and only the main stage was conducted [38].

Practice: The procedure is the same as the main stage (described below). The purpose of this stage is solely to practice and become familiar with the response method and the location of the keys on the keyboard, and it does not influence the final results.

##### Main stroop test procedure

In the main stage of the Stroop test, 480 color words (240 congruent and 240 incongruent) in the colors red, blue, yellow, and green are presented to the participant in a mixed and consecutive manner. Congruent words are those where the color of the word matches its meaning, such as the word “green” displayed in green. Incongruent words are those where the color of the word differs from its meaning, such as the word “green” displayed in red, blue, or yellow.

These 480 words are divided into 20 sets, each set comprising 24 congruent and incongruent words. The sets differ in the time interval between the stimuli, which includes 950, 850, 750, 650, and 550 milliseconds, applied randomly and equally to each set.

The participant’s task is to respond based solely on the color of the word, regardless of its meaning. The degree of inhibition or interference is calculated by subtracting the correct responses to incongruent stimuli from the correct responses to congruent stimuli. Additionally, the longer average response time to incongruent stimuli than congruent stimuli is another interference indicator.

The reliability of this test has been reported between 0.81 and 0.91. This main stage was evaluated at the beginning and end of the three shifts [41, 42].

#### Evaluation of cognitive performance (Working Memory Assessment)

Working memory refers to the limited amount of information that can be actively maintained and used in cognitive tasks, in contrast to long-term memory [43].

There are various methods to assess working (short-term) memory, one of the most common being the measurement of digit span. The digit span subtest of the Wechsler Intelligence Scale (for children and adults) measures working memory.

We used the computerized version of the Wechsler digit span subtest to assess visual working memory. Digit Span Test: “The digit span test was administered using a computerized version developed in-house.

The forward digit span test presents a series of 3 to 9 digits on the laptop screen, and the participant is required to select the digits in the same order using the mouse and keyboard. The backward digit span test presents a series of digits, and the participant must choose the digits in reverse order.

The Wechsler digit span test has a reliability of around 74% and a validity of over 90%. This assessment was conducted at the beginning and end of the three shifts [38, 44].

### Administration of cognitive tests

A meeting was held with the ICU matron and supervisors to explain the research objectives and the process of conducting the cognitive tests. We requested the supervisors to coordinate with the shift supervisors to inform the shift-working nurses about the research objectives and ensure their cooperation with the research team.

The cognitive tests were conducted in the nursing supervisor’s office, which provided a calm and conducive environment. After the shift-working nurses arrived at the supervisor’s office, they completed the informed consent form and the demographic questionnaire. Each nurse completed the cognitive assessments at both the beginning and the end of their respective shifts (morning, evening, and night). The data analysis employed mixed-effects models using analytical statistics, which were conducted with R software version 4. The significance level was at *p* < 0.05 for the present study.

## Results

### Data analysis

The data was analyzed using descriptive statistics, including mean, standard deviation, and frequency percentages. The Kolmogorov-Smirnov test was used to determine the normality of the data.

### Descriptive findings

#### Demographic information

The research findings showed that out of the 36 participants, 16.6% (6 individuals) were male, and 83.3% (30 individuals) were female nurses. The mean age and work experience were 37.38 and 13.33 years, respectively. Other information, including marital status, education level, employment status, age, and work experience, is presented in Table 1.

**Table 1:** Quantitative and qualitative demographic information of the participating nurses

Type of Information	Classification	Frequency	Percentage
Gender	Male	6	16.6
	Female	3	83.33
Marital Status	Single	10	27.78
	Married	26	72.22
Level of education	Bachelor	27	75
	Master and higher	9	25
Employment Status	Permanent	30	83.33
	Contractual	3	8.33
	Company-based	3	8.33
Type of Information		Mean	Standard Deviation
Age		37.389	6.308
Work Experience		13.333	6.234
Weekly Work Hours		55.194	11.749

### Mean Circadian Rhythm parameters

The mean and median of the circadian rhythm parameters are shown in Table 2. The mean circadian rhythm amplitude (LV) was 17.667, and the mean circadian rhythm stability (FR) was 13.583 (Table 2).

**Table 2:** Circadian rhythm

Type of Information	Lower and Upper Limits	Mean ± Standard Deviation	Median
Circadian Rhythm Range Languid/Vigorous(L-V)	6–30	17.66 ± 3.72	18
Circadian Rhythm Stability Flexible/Rigid (F-R)	5–25	13.58 ± 3.94	13

Regarding the comparison of Stroop test parameters (interference score), the results in Table 3 and the mixed-effects regression analysis indicate the following:

There is a statistically significant difference (*p* < 0.05) in the mean interference score between the beginning of the morning shift and the end of the night shift.

The mean interference score difference between the beginning of the evening shift and the end of the night shift and between the end of the evening shift and the end of the night shift was marginally significant. No statistically significant differences were observed in the other time points (*p* > 0.05).

**Table 3:** Comparison of interference scores at the beginning and end of work shifts

Variable	Shift Type	Statistic Value	*P*.value
**Interference score**	Beginning of morning shift vs. end of morning shift	3.13	0.204
	Beginning of morning shift vs. beginning of evening shift	0.19	0.937
	Beginning of morning shift vs. end of evening shift	0.27	0.910
	Beginning of morning shift vs. beginning of night shift	1.13	0.644
	Beginning of morning shift vs. end of night shift	4.94	0.046
	End of morning shift vs. beginning of evening shift	2.94	0.233
	End of morning shift vs. end of evening shift	2.86	0.246
	End of morning shift vs. beginning of night shift	2.00	0.417
	End of morning shift vs. end of night shift	1.80	0.464
	Beginning of evening shift vs. end of evening shift	0.08	0.973
	Beginning of evening shift vs. beginning of night shift	0.94	0.701
	Beginning of evening shift vs. end of night shift	4.75	0.055
	End of evening shift vs. beginning of night shift	0.86	0.726
	End of evening shift vs. end of night shift	4.66	0.059
	Beginning of night shift vs. end of night shift	3.80	0.124

Regarding the comparison of Wechsler test parameters (total correct responses), Table 4 and the mixed-effects regression analysis indicate the following:

There is a statistically significant difference (*p* < 0.05) in the mean total correct responses between:

- Beginning of the morning shift and the beginning of the night shift.
- Beginning of the morning shift and end of the night shift.
- End of the morning shift and beginning of the night shift.
- End of the morning shift and end of the night shift.
- Beginning of the evening shift and the beginning of the night shift.
- Beginning of the evening shift and end of the night shift.

No statistically significant differences were observed in the other time points (*p* > 0.05).

**Table 4:** Comparison of the average number of total correct responses at the beginning and end of work shifts

Variable	Shift Type	Statistic Value	*P*.value
Average total correct responses: V-Total	Beginning of morning shift vs. end of morning shift	0.16	0.880
	Beginning of morning shift vs. beginning of evening shift	0.19	0.860
	Beginning of morning shift vs. end of evening shift	1.66	0.133
	Beginning of morning shift vs. beginning of night shift	2.69	0.015
	Beginning of morning shift vs. end of night shift	2.55	0.022
	End of morning shift vs. beginning of evening shift	0.36	0.744
	End of morning shift vs. end of evening shift	1.83	0.099
	End of morning shift vs. beginning of night shift	2.86	0.010
	End of morning shift vs. end of night shift	2.72	0.014
	Beginning of evening shift vs. end of evening shift	1.47	0.185
	Beginning of evening shift vs. beginning of night shift	2.50	0.025
	Beginning of evening shift vs. end of night shift	2.36	0.034
	End of evening shift vs. beginning of night shift	1.02	0.354
	End of evening shift vs. end of night shift	0.88	0.422
	Beginning of night shift vs. end of night shift	0.13	0.900

Regarding the comparison of Wechsler test parameters (visual memory span), Table 5 and the mixed-effects regression analysis indicate the following:

There is a statistically significant difference (*p* < 0.05) in the mean visual memory span between the beginning of the morning shift and the beginning of the night shift.

The difference in the mean visual memory span between the beginning of the morning shift and the end of the night shift and between the beginning of the evening shift and the beginning of the night shift was marginally significant.

No statistically significant differences were observed in the other time points (*p* > 0.05).

**Table 5:** Comparison of visual memory capacity at the beginning and end of work shifts

Variable	Shift Type	Statistic Value	*P*.value
Visual memory span V-Span	Beginning of morning shift vs. end of morning shift	0.25	0.537
	Beginning of morning shift vs. beginning of evening shift	0.13	0.731
	Beginning of morning shift vs. end of evening shift	0.66	0.101
	Beginning of morning shift vs. beginning of night shift	0.88	0.029
	Beginning of morning shift vs. end of night shift	0.72	0.076
	End of morning shift vs. beginning of evening shift	0.11	0.783
	End of morning shift vs. end of evening shift	0.41	0.304
	End of morning shift vs. beginning of night shift	0.63	0.116
	End of morning shift vs. end of night shift	0.47	0.244
	Beginning of evening shift vs. end of evening shift	0.52	0.193
	Beginning of evening shift vs. beginning of night shift	0.75	0.065
	Beginning of evening shift vs. end of night shift	0.58	0.151
	End of evening shift vs. beginning of night shift	0.22	0.583
	End of evening shift vs. end of night shift	0.05	0.890
	Beginning of night shift vs. end of night shift	0.16	0.680

Here are the analytical findings:

### Impact of circadian rhythm on cognitive performance (attention and working memory)

The numbers 1 to 6 in the tables correspond to the following meanings:

This legend applies to the tables that follow. (Table 6)

**Table 6:** Codes related to the beginning and end of the shift

Shift time	Numbers
Beginning of morning shift	1
End of morning shift	2
Beginning of evening shift	3
End of evening shift	4
Beginning of night shift	5
End of night shift	6

Regarding the comparison of circadian rhythm dimensions in the morning, evening, and night shifts with congruent response time, the results in Table 7 and the mixed-effects model analysis indicate the following:

There is a statistically significant difference (*p* < 0.05) in the relationship between circadian rhythm amplitude and stability with congruent response time between the morning-evening and morning-night shifts.

No statistically significant differences were observed in the other time points (*p* > 0.05).

It means that with a one-unit increase in circadian rhythm stability and amplitude, the congruent response time decreases in the evening and night shifts compared to the morning shift.

**Table 7:** Comparison of circadian rhythm dimensions in morning, evening, and night shifts with the amount of response time in harmony

Variable	Shift Type	Statistic Value	*P*.value
Circadian rhythm amplitude (L-V)	L-V(2 − 1)-(3–4)	-568.06	< 0.001
	L-V(2 − 1)-(6 − 5)	-610.57	0
	L-V(3–4)-(6 − 5)	-42.51	0.966
Circadian rhythm stability (F-R)	F-R(2 − 1)-(3–4)	-568.68	0
	F-R(2 − 1)-(6 − 5)	-587.26	0
	F-R(3–4)-(6 − 5)	-18.57	0.995

Regarding the comparison of circadian rhythm dimensions in the morning, evening, and night shifts with incongruent response time, Table 8 and the mixed-effects model analysis indicate the following:

There is a statistically significant difference (*p* < 0.05) in the relationship between circadian rhythm amplitude and stability with incongruent response time between the morning-evening and morning-night shifts.

No statistically significant differences were observed in the other time points (*p* > 0.05).

It means that with a one-unit increase in circadian rhythm stability and amplitude, the incongruent response time decreases in the evening and night shifts compared to the morning shift.

**Table 8:** Comparison of circadian rhythm dimensions in morning, evening and night shifts with inconsistent response time

Variable	Shift Type	Statistic Value	*P*.value
Circadian rhythm amplitude (L-V)	L-V(2 − 1)-(3–4)	-554.74	0.001
	L-V(2 − 1)-(6 − 5)	-625.33	0
	L-V(3–4)-(6 − 5)	-70.58	0.921
Circadian rhythm stability (F-R)	F-R(2 − 1)-(3–4)	-558.77	0.001
	F-R(2 − 1)-(6 − 5)	-598.89	0
	F-R(3–4)-(6 − 5)	-40.11	0.977

Regarding the comparison of circadian rhythm dimensions in the morning, evening, and night shifts with the interference score, Table 9 and the mixed-effects model analysis indicate that there was no statistically significant difference (*p* > 0.05) between circadian rhythm amplitude and stability with the interference score across the shifts. The findings did not show a significant relationship between the circadian rhythm dimensions (amplitude and stability) and the Stroop interference score across the different shifts.

**Table 9:** Comparison of circadian rhythm dimensions in morning, evening and night shifts with interference score

Variable	Shift Type	Statistic Value	*P*.value
Circadian rhythm amplitude (L-V)	L-V(2 − 1)-(3–4)	-1.25	0.999
	L-V(2 − 1)-(6 − 5)	19.33	0.569
	L-V(3–4)-(6 − 5)	2.59	0.606
Circadian rhythm stability (F-R)	F-R(2 − 1)-(3–4)	-1.59	0.998
	F-R(2 − 1)-(6 − 5)	19.94	0.556
	F–R(3–4)-(6 − 5)	21.54	0.585

Regarding the comparison of circadian rhythm dimensions in the morning, evening, and night shifts with visual memory span, Table 10 and the mixed-effects model analysis indicate a statistically significant difference (*p* < 0.05) between circadian rhythm amplitude and stability with the visual memory span in the morning-evening and morning-night shifts. It means that with a one-unit increase in circadian rhythm stability and amplitude, the visual memory span decreases in the evening and night shifts compared to the morning shift. (Table 10)

**Table 10:** Comparison of circadian rhythm dimensions in morning, evening, and night shifts with visual memory span

Variable	Shift Type	Statistic Value	*P*.value
Circadian rhythm amplitude (L-V)	L-V(2 − 1)-(3–4)	-9.42	0.006
	L-V(2 − 1)-(6 − 5)	-7.75	0.03
	L-V(3–4)-(6 − 5)	1.67	0.887
Circadian rhythm stability (F-R)	F-R(2 − 1)-(3–4)	-9.48	0.004
	F-R(2 − 1)-(6 − 5)	-7.59	0.036
	F-R(3–4)-(6 − 5)	1.89	0.86

## Discussion

This study investigated the impact of circadian rhythm disturbances on the cognitive performance of ICU nurses in Qazvin City, Iran. Regarding circadian rhythm status among the shift-working ICU nurses, the mean circadian rhythm amplitude was 17.667, which is less than 22.5 and indicates that these individuals belong to the “morning-active” chronotype. For these individuals, overcoming sleepiness is relatively easy.

The mean circadian rhythm stability was 13.583, which is less than 18.75 and classifies these individuals as having a “rigid” circadian rhythm type. These individuals have lower capability for shift work employment.

Most participants had a “morning-active, but rigid” personality type. This part of the study is consistent with the findings of Yuex et al., who examined the impact of circadian rhythm and sleep quality on occupational burnout among shift-working nurses, and the study by Wu et al., which investigated the mediating effect of circadian rhythm amplitude between work stress and sleep quality in shift-working nurses [45, 46].The results also align with the study by Jafari Rudbandi, which explored the relationship between circadian rhythm amplitude, stability, sleep quality, and sleepiness in shift-working nurses and healthcare workers [47]. However, our findings contradict the study by Daneshvar et al., which examined the circadian rhythm type of nursing students [48].

Regarding cognitive performance, one sub-scale for evaluating the level of attention in nurses is the interference score. Our results showed that the mean interference score increased after all three work shifts (morning, evening, and night), but the increase was more significant at the end of the night shift.

The difference in the mean interference score was statistically significant only between the beginning of the morning shift and the end of the night shift. The difference in the mean interference score between the beginning of the evening shift and the end of the night shift and between the end of the evening shift and the end of the night shift was marginally significant.

An increase in the interference score indicates that the number of correct incongruent responses was lower than the correct congruent responses, meaning that the number of errors increased and attention decreased. Our findings showed that the lowest mean interference score was at the beginning of the morning shift, and the highest was at the end of the night shift. Therefore, attention was high at the beginning of the morning shift and low at the end of the night shift.

Several studies, consistent with the present study, have shown that attention is disrupted and decreases during night shifts. Donmez-Dil et al. found that the level of attention during the night shift was significantly lower than on the day shift. The systematic review by de Cordova et al. on shift work, increased errors, and decreased performance during night shifts showed that individuals have poorer health, which may lead to errors and reduced performance at work. Similarly, the study by Esmaili et al. on the effect of shift work on working memory, attention, and response time in nurses found the lowest interference score at the beginning of the morning shift and the highest at the end of the night shift. Our findings are consistent with these studies [38, 49, 50].

Decreased attention can affect the ability to perform specific tasks that require special attention, such as medication administration or writing important reports. Significant changes in interference time were observed at the end of the morning and night shifts. Increased interference scores lead to longer response times to incongruent stimuli, slowing the decision-making process, which is particularly important in emergencies [38].

Our findings regarding the working memory showed that the highest working memory score was observed at the beginning of the night shift, and the lowest (weakest) was at the beginning of the morning shift. Comparing the beginning and end of each shift also showed that the working memory score at the end of the night shift was non-significantly lower than the beginning of the night shift.

Therefore, some cognitive and executive functions, such as reasoning and comprehension, may be impaired due to reduced working memory, hurting critical nursing tasks. The results of this part are somewhat consistent with the study by Tadevinac et al., which examined the effect of age and individual sleep characteristics on the cognitive performance of anesthesia residents after a 24-hour shift, the systematic review by de Cordova et al. on shift work, increased errors, and decreased performance during night shifts, and the study by Leso et al. on the impact of shift work on cognitive performance [16, 26, 50].

The results of the study on the impact of circadian rhythm dimensions on cognitive performance (attention and working memory) showed that with a one-unit increase in circadian rhythm stability and amplitude, the visual memory span, congruent response time, and incongruent response time decreased in the evening and night shifts compared to the morning shift. The results indicate that visual memory and attentional capacity, as measured by congruent and incongruent response times, were better in the morning shift than the evening and night shifts.

The findings suggest that nurses’ cognitive performance was higher in the morning shift, with better attention and working memory. These results are consistent with the studies by Esmaili et al., Malti et al., and Zijlstra et al. [38, 39, 51]. The review by Xiang et al. stated that circadian rhythm generally affects cognitive functions, but their findings that individuals had the best performance in attention and working memory in the afternoon and the worst in the early morning contradict our results [52].

The nature of the nursing profession requires high levels of attention and focus in caring for patients. Therefore, it is natural that changes in mental abilities are related to the type and quality of nursing errors, where a decrease in attention and focus can lead to the transformation of near-misses into actual errors or errors without observable effects on the patient, such as prolonged nursing care or transfer to specialized units [53].

The Stroop stimuli focus on measuring selective attention to the color of the word without considering its meaning. This method targets the process of attentional control (color naming) and the automatic control process (word meaning). The presentation of different colors (green, blue, yellow, and red) in the form of congruent and incongruent words creates the Stroop interference effect, where the conflict between the color and the word meaning increases the complexity of the stimulus analysis and response selection process [54].

Complex stimuli have longer reaction times than simple stimuli, as initiating responses is more difficult, leading to increased deliberation and decision-making for new stimuli. This incongruent condition of the Stroop effect increases the complexity of the stimulus analysis process at the response initiation stage, as the target stimulus must be selected from among the distracting factors (color-word incongruence). On the other hand, congruent stimuli can facilitate the stimulus analysis process in the response initiation stages, as the background color is consistent with the meaning, simplifying the response selection process. Therefore, the response time to congruent stimuli is faster than the incongruent condition [55].

Shift workers need complete attention and alertness during their shifts to perform their duties effectively, and they also require time to compensate for the lack of rest, which decreases over time, leading to disruptions in mental performance and the manifestation of the consequences of shift work [56, 57]. Ruggiero et al. showed that scheduled naps during night shifts can reduce nighttime sleepiness and improve sleep-related performance deficits [58]. Strategies to reduce sleep deprivation and improve attention during the night shift may reduce the rate of night-shift errors. Studies have shown that moderate exercise for 30 min, napping, and moderate caffeine consumption before the night shift can increase alertness. However, consuming simple sugars or prolonged strenuous exercise can increase sleepiness [59].

## Limitations

Time constraints of nurses for completing the questionnaire: Healthcare personnel, especially nurses, are often overburdened due to a shortage of human resources relative to the volume of activities, and they are often unwilling to complete questionnaires. To address this issue, the researchers coordinated with the nurses in advance to ensure that they had sufficient time to complete the questionnaires.

Generalizability of the results: Since this study was conducted among nurses in academic medical centers, caution should be exercised when generalizing the findings to nurses in non-academic and private hospitals.

The absence of sleep status measurement may be regarded as another limitation of this study. It is advisable for future research to address this issue by incorporating assessments of sleep quality.

## Conclusion

The circadian rhythm was related to certain aspects of nurses’ cognitive performance. Some sub-scales of cognitive performance significantly decreased in the evening and night shifts than the morning shift. As a result, the likelihood of errors during the evening and night shifts may increase. The findings also indicate that the cumulative effect of the disruptions caused by the individual’s circadian rhythm disturbances can manifest as a reduction in cognitive performance.

## Data Availability

The datasets used and/or analyzed during the current study available from the corresponding author on reasonable request. The entire dataset is in Farsi language. The Data can be available in English language for the readers and make available from the corresponding author on reasonable request..

## Appendix

### Average scores of the Stroop Test (attention):

Tables 4, 5, 6, 7, 8, 9, 10 and 11 present the mean interference scores from the Stroop test at the beginning and end of each of the three shifts. Table 12 displays the mean visual memory span scores at the beginning and end of work shifts. The results indicate that the highest interference scores were recorded at the end of the night shift and the end of the morning shift.

**Table 11:** Interference scores at the beginning and end of work shifts

Statistic value	Testing Time	Mean ± SD
Interference Score	Beginning of morning shift	4.66 ± 6.84
	End of morning shift	7.80 ± 8.46
	Beginning of evening shift	4.86 ± 6.25
	End of evening shift	4.94 ± 7.33
	Beginning of night shift	5.80 ± 18.12
	End of night shift	9.61 ± 13.71

**Average Scores of Wechsler Test Parameters (Visual Memory Span)**.

**Table 12:** Displays the mean visual memory span scores at the beginning and end of work shifts

Statistic value	Testing Time	Mean ± SD
**Visual Memory Span** (V-Span)	Beginning of morning shift	5.472 ± 1.844
	End of morning shift	5.722 ± 1.542
	Beginning of evening shift	5.611 ± 1.712
	End of evening shift	6.139 ± 1.606
	Beginning of night shift	6.361 ± 2.031
	End of night shift	6.194 ± 1.969
